# Complete Genome Sequence of Bacillus amyloliquefaciens KNU-28 Isolated from Peach Leaves (Prunus Persica [L.] Batsch)

**DOI:** 10.1128/mra.00734-22

**Published:** 2022-09-19

**Authors:** Min-Ji Kim, TaeHyung Park, Minsoo Jeong, GyuDae Lee, Da-Ryung Jung, Jae-Ho Shin

**Affiliations:** a Department of Applied Biosciences, Kyungpook National University, Daegu, Republic of Korea; b Department of Integrative Biology, Kyungpook National University, Daegu, Republic of Korea; c Next Generation Sequencing (NGS) Core Facility, Kyungpook National University, Daegu, Republic of Korea; University of Arizona

## Abstract

This study presented the complete genome sequence of B. amyloliquefaciens KNU-28 isolated from the leaves of the peach (Prunus persica [L.] Batsch). The genome of this strain comprised one chromosome with 4,238,926 bp and 45.9% of GC content.

## ANNOUNCEMENT

Bacillus amyloliquefaciens is a Gram-positive, aerobic, and rod-shaped bacterium that is used in various environments as anti-pathogenic bacteria (1–3). To investigate its anti-fungal potential, we obtained the genome sequence of B. amyloliquefaciens KNU-28, isolated from the leaves of the peach (Prunus persica [L.] Batsch), in Apo-eup, Gimcheon-si, Gyeongsangbuk-do, Republic of Korea (128.25°N, 36.184°E). Briefly, 0.25 g of the peach leaves were homogenized using BioMasher-III (Optima Inc., Japan), suspended in 0.85% of NaCl solution, and then plated onto potato dextrose agar (PDA; Difco, USA) plates. The single colony of this strain was inoculated in a potato dextrose broth (PDB) medium and incubated at 30°C for 18 h at 200 rpm.

The genomic DNA was extracted using Wizard Genomic DNA purification kit (Promega, USA) following the manufacturer’s protocol. The extracted DNA was quantified by Qubit 3.0 fluorometer (Thermo Fisher Scientific, USA). In addition, the quality of the genomic DNA was measured by Nanopore One Spectrophotometer (Thermo Fisher Scientific, USA). The genomic DNA was not sheared by specific size for the sequencing library preparation. The sequencing library preparation was performed using the Ligation Sequencing kit SQK-LSK109 (Oxford Nanopore Technologies) and NEBNext Companion Module for Oxford Nanopore Technologies Ligation Sequencing kit (New England BioLabs, USA) following the manufacturer’s protocol. The prepared sequencing library was loaded into the flow cell FLO-MIN111 (R10.3, Oxford Nanopore Technologies). The long-read sequencing was performed using Nanopore MinION at the next-generation sequencing (NGS) core facility, Kyungpook National University, South Korea. FASTQ files of the sequencing data were generated from base calling using Guppy (v.4.4.1) (Ubuntu 18 GPU, GeForce GTX 1660) (4). The sequences with Phred score lower than 7 were excluded from downstream analysis. After quality trimming, 1,655,695,148 reads were obtained with 5,139 bp of N_50_. Flye (v.2.9) was used for *de novo* assembly. Genome assembly was conducted with default parameters, excepting genome size option (option: –nano-raw –genome-size 4.0) (5).

**FIG 1:**
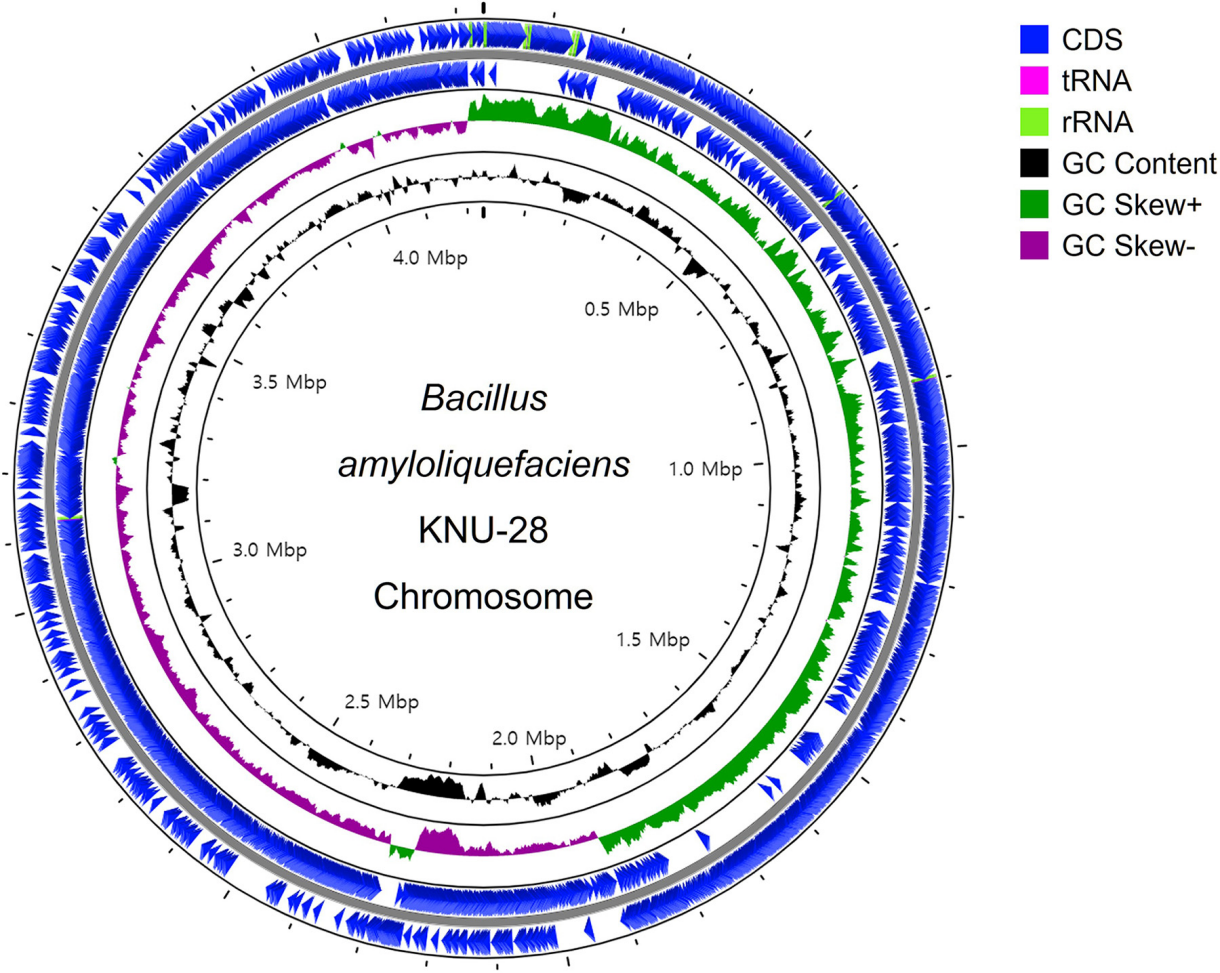
The circular chromosome sequence of B. amyloliquefaciens KNU-28. The visualization of the genome map was performed using CGView.

The genome of B. amyloliquefaciens KNU-28 consists of 4,238,926 bp (1 contig; *N*_50_, 4,238,926 bp) with 387× coverage. After getting the contig of the strain, the completion of the assembly was confirmed by a dot plot generated from Gepard (6). The complete genome sequence was visualized by CGView (Fig. 1) (7). Furthermore, genome annotation of B. amyloliquefaciens KNU-28 was conducted using NCBI PGAP (8) and the RAST server (9). The genome annotation with the NCBI PGAP identified 3,309 protein-coding genes, 28 rRNAs, 86 tRNAs, 5 ncRNAs, and 873 pseudogenes.

### Data availability.

The complete genome sequence of B. amyloliquefaciens KNU-28 has been deposited in the DDBJ/ENA/GenBank database under the accession number CP101315.1. The raw sequencing data can be accessed with the accession number (SRR20305873).
